# Trophic factors and cell therapy to stimulate brain repair after ischaemic stroke

**DOI:** 10.1111/j.1582-4934.2012.01575.x

**Published:** 2012-09-26

**Authors:** María Gutiérrez-Fernández, Blanca Fuentes, Berta Rodríguez-Frutos, Jaime Ramos-Cejudo, María Teresa Vallejo-Cremades, Exuperio Díez-Tejedor

**Affiliations:** a Department of Neurology and Stroke Centre, La Paz University Hospital Neuroscience Area of IdiPAZ (Health Research Institute) Autónoma University of MadridMadrid, Spain; b Neuroscience and Cerebrovascular Research Laboratory, La Paz University Hospital Neuroscience Area of IdiPAZ (Health Research Institute) Autónoma University of MadridMadrid, Spain

**Keywords:** brain plasticity, brain protection, brain repair, trophic factors, stem cell therapy

## Abstract

Brain repair involves a compendium of natural mechanisms that are activated following stroke. From a therapeutic viewpoint, reparative therapies that encourage cerebral plasticity are needed. In the last years, it has been demonstrated that modulatory treatments for brain repair such as trophic factor- and stem cell-based therapies can promote neurogenesis, gliogenesis, oligodendrogenesis, synaptogenesis and angiogenesis, all of which having a beneficial impact on infarct volume, cell death and, finally, and most importantly, on the functional recovery. However, even when promising results have been obtained in a wide range of experimental animal models and conditions these preliminary results have not yet demonstrated their clinical efficacy. Here, we focus on brain repair modulatory treatments for ischaemic stroke, that use trophic factors, drugs with trophic effects and stem cell therapy. Important and still unanswered questions for translational research ranging from experimental animal models to recent and ongoing clinical trials are reviewed here.

- Brain repair after ischaemic stroke
- Trophic factor-based therapies
  – Experimental animal models
  – Trophic factors
  – Clinical studies
- Stem cell therapies
  – Experimental animal models
  – Clinical studies
- Last comments

## Brain repair after ischaemic stroke

Protective therapies that focused on saving just the neural cells instead of protecting all the components of the neurovascular unit have consistently failed [1]. Rather than being simple, recovery from ischaemia is a complex and highly dynamic process that includes not only injury and response signals within the lesions but also active self-repair processes that occur in the whole organ [2–5] and that should be precisely synchronized for tissue remodelling. Neurogenesis, gliogenesis, oligodendrogenesis, synaptogenesis and angiogenesis are brain repair-associated processes that are activated following stroke. In recent decades, animal models of cerebral ischaemia and clinical research have demonstrated how brain repair processes can be actively modulated by the administration of both trophic factors and stem cells.

**Fig 1:**
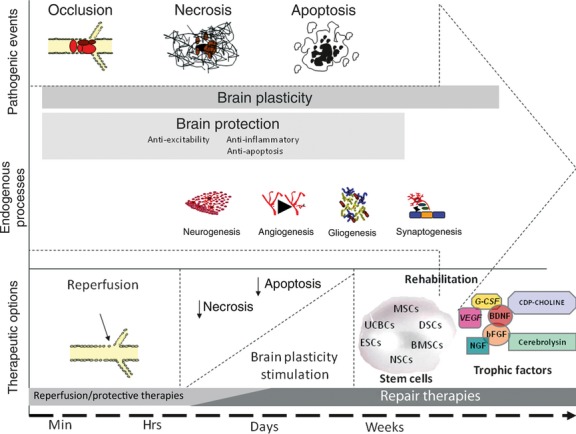
Pathogenic mechanisms and therapeutic options in cerebral infarct. Time line for the mechanisms and therapy involved in endogenous protection and brain protection-repair after ischaemic stroke. MSCs: mesenchymal stem cells; UCBCs: umbilical cord blood cells; DSCs: dental stem cells; ESCs: embryonic stem cells; BMSCs: bone marrow stem cells; NSCs: neural stem cells; G-CSF: granulocyte colony-stimulating factor; VEGF: vascular endothelial growth factor; BDNF: brain-derived neurotrophic factor; NGF: nerve growth factor; bFGF: basic fibroblast growth factor; IGF-1: insulin growth factor-1; EPO: erythropoietin.

We should first consider that protection and repair mechanisms are activated and work together from the very beginning of cerebral ischaemia (Fig. 1). The accompanying hypoxia and glucose deprivation, cell death programs and immunological events of ischaemia are initiated to remove damaged cells and tissue debris and to prepare injured areas for repair processes [6–8] and as a response to injury, transcriptional programs associated with axonal sprouting, survival and myelin formation are activated and maintained from the very beginning [9, 10]. Research is now focused on how to modulate these processes to preserve all the structures that make up the neurovascular unit, including microvessels and pericytes (vascular protection), neurons and their axons (neuroprotection), astrocytes and other supportive cells such as oligodendroglia [11].

Synchronized events after damage may allow initial deleterious signals to transition into beneficial effects and recovery [12]. During the early acute phase, blood-brain barrier disturbances predominate and matrix proteases like MMP-4 or MMP-9 are essential for neurovascular remodelling, while during the late phase, other processes, such as angiogenesis, may provide the critical substrate for remodelling. Understanding how neurovascular signals and substrates make the transition from initial injury to angiogenic recovery is important for obtaining new therapeutic options as a cerebral infarct is a highly complex condition whose effects might extend beyond time (time since the ischaemic insult) and location (communication between brain ischaemic regions and healthy areas).

**Fig 2:**
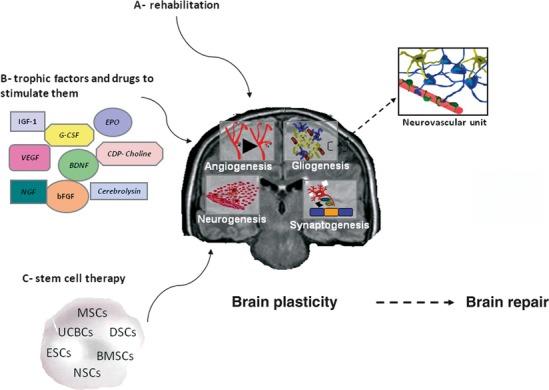
Brain repair therapies through brain plasticity enhancement. Mechanisms underlying cerebral plasticity associated with repair processes. G-CSF: granulocyte colony-stimulating factor; VEGF: vascular endothelial growth factor; BDNF: brain-derived neurotrophic factor; NGF: nerve growth factor; bFGF: basic fibroblast growth factor; IGF-1: insulin growth factor-1; EPO: erythropoietin; MSC: mesenchymal stem cells; UCBC: umbilical cord blood cells; DSC: dental stem cells; ESC: embryonic stem cells; BMSC: bone marrow stem cells; NSC: neural stem cells.

Trophic factors, stem cell therapy and rehabilitation have all been shown to exert potential therapeutic effects by modulating brain repair- associated mechanisms (Fig. 2). In experimental animals, increased levels of neurogenesis, gliogenesis, oligodendrogenesis and angiogenesis accompanied with better functional recovery have been widely reported after treatment [13, 14]. Such promising pre-clinical results have led to multiple clinical trials in the last years. In this review, we will discuss recent reports from both pre-clinical and clinical studies that raise important new questions and concerns for further advances in the field.

## Trophic factor-based therapies

The discovery of nerve growth factor (NGF) in the 1950s by Levi-Montalcini and Hamburger [15] opened a promising new era in physiology in which the growth-factor induced regeneration of damaged tissues seemed to be possible and its therapeutic potential has been explored in both experimental animals and clinical trials (Table 1). As could expected, new hopes and fundamental questions have emerged over the last years. This approach could be based on the direct administration of trophic factors, and of drugs with trophic effects.

**Table 1:** Main results of therapeutic studies with trophic factors or drugs with trophic effects in cerebral infarct animal models and human clinical trials

	Animal models	Clinical trials
Trophic factors		
Basic fibroblast growth factor (bFGF)	Promotes neurogenesis [18] Enhances functional recovery and stimulates progenitor cell proliferation [19]	Phase III (286 patients). Prematurely stopped [53]
Brain-derived neurotrophic factor (BDNF)	Cellular and functional recovery [21] Protects and promotes nerve fibreregeneration [22] Promotes prostacyclin biosynthesis [23]	No studies
Vascular endothelial growth factor (VEGF)	Reduces neuronal cell death, increases angiogenesis and vascular permeability [116, 117] reduces infarct volume, improves behavioural recovery [30]	No studies
Erythropoietin (EPO)	Reduces infarct size and improves neurobehavioral deficits [41]	Safety: open label (13 patients); Efficacy: double-blind randomized proof of concept trial (40 patients): Improvement in neurological outcome, and smaller lesion size [55] Phase II/III (522 patients): negative results and safety concerns [56]
Granulocyte colony-stimulating factor (G-CSF)	Promotes new blood vessel formation, has anti-inflammatory, anti-excytotoxic, neuroprotective properties [43] and survival-enhancing capacity and effects on functional outcome [44]	Safety: Phase IIb (60 patients): [58] Safety and efficacy: AXIS-2 Trial finished. Results not yet published
EPO + G-CSF	Enhances angiogenesis and tissue plasticity, leading to greater functional recovery [45]	No studies
Drugs with trophic effects		
CDP-choline (citicoline)	Increases neuronal plasticity and contributes to sensorimotor function recovery [48] Promotes protective and repair mechanisms [46, 47, 49]	Efficacy and safety: Individual pooled data analysis [61] Efficacy: Phase III (ICTUS Trial; 2078 patients) finished 62. Results not yet published
Porcine brain derived peptide (cerebrolysin)	Reduces infarct volume and improves recovery [50] with increased neurogenesis [51], efficacy in neurological recovery, reduction of neuronal death, increased cell proliferation and decreased inflammatory response [52]	Safety and efficacy: Phase II clinical trial (146 patients) [63] and Cochrane Syst Rev [64]: not enough evidence for efficacy. No safety concerns Safety and efficacy: Phase IV Clinical trial finished: CASTA (1070 patients) safety confirmed; possible efficacy in more severe strokes [66]

A non-systematic selection of the main results of therapeutic studies with trophic factors or drugs with trophic effects in animal models and clinical trials of cerebral ischaemia is provided. The reference number for each study is shown in brackets. Information from ongoing clinical trials can be consulted in the PubMed (http://www.ncbi.nlm.nih.gov/pubmed/) and clinical trials (http://clinicaltrials.gov/) databases.

### Experimental animal models

#### Trophic factors

The reported number of biological modulatory molecules that mediate in brain repair is high and ever-growing. Besides NGF, which has been reported to improve cholinergic function, stimulate axonal growth, cerebral perfusion and neurogenesis by stimulating proliferation through tyrosine kinase receptor signalling [16, 17]*,* the administration of other factors, like basic fibroblast growth factor (bFGF), has been shown to promote neurogenesis in both intact and ischaemic brain [18]. Indeed, intracysternal administration 1 day after experimental stroke in rats has been shown to stimulate progenitor cell proliferation in the subventricular zone (SVZ) and dentate gyrus (DG), important areas for the development of new neurons in the adult brain [19]. While higher levels seem to be required after damage [9], it is important to emphasize that trophic factors not only act in disease but also under normal conditions to maintain tissue homeostasis. This has been reported in brain-derived neurotrophic factor (BDNF) signalling, impairment of which may cause progressive neuronal dysfunction in animal models [20]. In this sense, intravenous administration of BDNF during the 5 days following cortical photothrombotic stroke is associated with enhanced migration of progenitor cells from the SVZ and increased neurogenesis in the DG on DCX- and NeuN-stained slices [21].

How can brain repair be modulated by the action of factors like BDNF? Although still unclear, white matter glial cells have been reported to play a key role in protecting and promoting the regeneration of nerve fibres by producing BDNF itself [22]. Also, prostacyclin, an important hormone released in response to vascular damage is stimulated around cerebral arteries when this factor is present [23]. From a genetic perspective, it is known that BDNF can activate NF-kB through the TrkB-PI3-Kinase-Akt pathway [24] and that this activation leads to the downstream activation of genetic programs that contribute to protecting cells from stress conditions such as serum starvation, glutamate toxicity or ischaemia [25], all of which occur at the beginning of the ischaemic insult.

It bears mentioning that trophic factors not only enhance single processes like neurogenesis, but they also exert pleiotropic effects on other biological pathways such as vascular function, immune cell function or cell death. In this sense, it was recently reported that the preserved neuronal loss and reduced number of TUNEL-positive cells after intranasal administration of BDNF might also be due to modulation of local inflammation by this factor, which would reduce tumour necrosis factor-α (TNF-α) levels and augment those of interleukin (IL)-10 [26].

However, in addition to all of this pleiotropic interplay, the activity of most of these factors within the brain under ischaemic conditions is not clear. After the hypoxic insult, many hypoxia-response genes such as HIF-1alpha are upregulated, triggering downstream changes in other interacting genes such as vascular endothelial growth factor (VEGF), which is the key gene for the angiogenesis induced in penumbral regions of the brain. This angiogenesis is known to depend on several factors including VEGF, VEGFR2, Angiopoietins 1 and 2 and its Tie2 receptors [27]. In a recent study, inhibition of VEGF receptor 2 after ischaemia worsened injury and also affected cell death patterns with a shift from apoptosis to a necrosis phenotype [28]. In many other studies in which VEGF was administrated following stroke, the growth factor was shown to enhance brain repair processes [29, 30]. For all these reasons VEGF and its signalling of vasculogenesis has attracted much interest in recent years, revealing that neurogenesis is not the only process that responds to trophic factor therapy among possible brain repair therapies. Indeed, some trophic factors such as insulin growth factor-1, which has been reported to promote recovery after stroke [31–33], exert their activity in different routes by enhancing endothelial function, regulating apoptosis and having anti-inflammatory properties instead of just affecting neurogenesis [34, 35].

Another process that is modulated by brain repair therapies is myelin formation. Again, we emphasize the importance of connections between elements of the different pathways involved in brain repair after ischaemia. Recent publications have suggested connections within signal transduction pathways between elements such as Lingo-1 and epidermal growth factor [36]. Given that Lingo1 antibodies can promote recovery from demyelinating disease in animal models [37], trophic factors that might modulate Nogo-A or Lingo1 activities may offer interesting possibilities for brain repair. Important inhibitors of axonal remodelling, such as Nogo-A, are augmented after cerebral ischaemia [38] and their inhibition through viral-mediated RNAi enhances axonal connectivity [39]; therefore, strategies that enhance myelin formation and axonal remodelling through trophic factors are a possible way forward in stroke research.

Other possible treatment approaches are blood-mobilizing drugs like erythropoietin (EPO) [40–42] or Granulocyte colony-stimulating factor (G-CSF), which have been shown to have positive results in animal models [43, 44]. Interestingly, higher levels of neovascularization and endogenous stem cell biological activity were observed when these factors were combined in a recent study [45]. In light of these results, an open question in trophic factor therapy is whether augmented efficacy could be obtained by applying these factors in specific combinations instead of using any one factor alone.

On the other hand, some other drugs with trophic effects*,* like CDP-choline, which have been attributed with a protective role [46, 47], have come under investigation. Although its mechanisms of action are unknown, it is thought that CDP-choline improves both the structural integrity and functionality of the neuronal membranes, and this may in turn assist membrane repair [48]. Experimental animal studies have demonstrated that CDP-choline not only promotes protective mechanisms (decreasing gliosis and cell death) but also seems to stimulate repair (increasing endogenous cellular proliferation, angiogenesis and synaptogenesis) [49]. Another drug that has been attributed with a protective role is Cerebrolysin, which has exhibited trophic properties when applied 24 and 48 hrs after stroke in animal models of ischaemia [50]. Indeed, while infarction volume does not seem to be substantially reduced with this treatment, the functional outcome is improved and proliferation, migration and survival of neuroblasts, especially in the peri-infarct area, have been thought to contribute to the observed results [51, 52].

### Clinical studies

Some of the trophic factors mentioned above, including bFGF, EPO and G-CSF, have been tested in controlled human clinical trials (Table 1). Unfortunately and in contrast with the experimental studies, the results have been mixed. As an example, a phase III clinical trial for intravenous bFGF administration for acute ischaemic stroke was stopped because an interim analysis of efficacy data predicted too small a chance of demonstrating a statistically significant benefit. In addition, unexpected peripheral side effects including leucocytosis and decreased blood pressure were reported in the treated group [53]. Nevertheless, new trials are still being considered as the pre-clinical data continue to justify further controlled clinical research with larger cohorts of patients [54].

In the case of EPO, the results are also inconclusive. While an initial small-size proof of concept trial performed in acute stroke patients reported an improvement in stroke outcome at 1 month and significantly smaller lesion size in the treated group than in controls without relevant side effects [55], a larger phase II/III study with EPO ended with negative results and safety concerns [56]. The possibility that some of these factors could be especially interesting for specific stroke subtypes is also not clear at the moment. Peripheral blood mobilization factors like G-CSF have been tested in humans and have been found to be safe [57, 58]. In addition, an exploratory analysis has suggested dose-dependent beneficial effects from G-CSF treatment in patients who had large baseline diffusion-weighted image lesions, supporting further investigation and repeated trials with larger cohorts of patients [59]. An AXIS-II trial investigating the safety, tolerability and effect of G-CSF in acute ischaemic stroke patients has recently completed recruitment but results have not yet been published 60.

Based on their pre-clinical results, other drugs with trophic effects*,* specifically CDP-choline and Cerebrolysin, have also been tested in clinical trials. A meta-analysis of pooled data collected from several small phase III trials, concluded that CDP-choline was reported to be safe and present a certain efficacy. In patients with moderate to severe ischaemic stroke, oral CDP-choline for 6 weeks increased the global odds of recovery at 3 months by 33% compared with the placebo [61] and this is being studied more thoroughly in a larger clinical phase III trial (ICTUS) 62, which has recently completed recruitment with results to be published soon 60. In the case of Cerebrolysin, a randomized placebo-controlled trial (146 patients) showed a significant improvement of cognitive function of patients treated with Cerebrolysin, but without a significant effect on neurological or functional outcome [63]. A systematic Cochrane review reported not enough evidence to evaluate the effect of cerebrolysisn on survival and dependency in acute ischaemic stroke with no safety concerns [64], a large double-blind placebo-controlled randomized phase III clinical trial (1070 patients) conducted in Asiatic patients (CASTA), confirmed its safety and suggested a benefit for the group with more severe strokes [65, 66]. Thus, it could be interesting to continue the research development of this drug.

In summary, to date there is insufficient knowledge of efficacy of trophic factors in ischaemic stroke based on clinical trials, and the publication of the results of the AXIS-II trial should give more information. On the other hand, research on drugs with trophic effects has demonstrated the safety of CDP-choline and of Cerebrolysin, and suggests some efficacy in acute ischaemic stroke.

## Stem cell therapies

As well as immune-modulation and substitution of damaged areas under certain conditions [27, 67], the available evidence supports the concept that stem cells assist recovery by modulating brain repair processes, including neurogenesis, gliogenesis, synaptogenesis and angiogenesis. While the molecular events underlying these processes are mostly unknown, it has been suggested that stem cells are capable of secreting trophic factors (VEGF, bFGF, BDNF) [68], in response to repair processes amplifying their levels in the brain. After culture in *ex-vivo* experiments, trophic interactions between MSCs and ischaemic brain have led to increased production of trophic factors including BDNF, VEGF or HGF (hepatocyte growth factor) [69] and it is known that stem cells express receptors that might allow these interactions [70, 71]. Furthermore, it has been discovered that stem cell transplantation is more effective when implanted cells are derived from stroke animals than when harvested from controls [72]. This recent report supports the hypothesis of trophic interactions between damaged brain and stem cells under ischaemic conditions that would prepare stem cells to exert their positive function. Interestingly, if these trophic interactions exist, another treatment strategy might be to combine stem cell therapy methods with trophic factor pre-treatment *in vitro* before their application. Under this perspective, stem cells genetically modified to overexpress specific trophic factors might enhance neuronal differentiation and survival [73]; in addition, gene modification of MSCs using viral vectors or RNA-based techniques may be a key to obtaining enhanced expression of specific desired factors (*i.e*. FGF-2) in comparison with ‘wild type’-MSC transplantation [74].

Four major aspects will be reviewed below, concerning: (i) stem cell sources; (ii) the type of cell transplant, based on cell source; (iii) the time of administration or therapeutic window; and finally, (iv) the most suitable administration route for its clinical translation.

Looking at the wide range of stem cell sources, cerebral ischaemia can be treated using different types of cells from different origins (see Table 2). Enhanced function has been reported with different cell populations under different experimental conditions [75, 76]. Although a variety of conditions have been proposed for cell therapy, there is still no proven stem cell-based approach for stroke treatment and substantial symptomatic relief has not yet been demonstrated in patients [67, 76, 77].

**Table 2:** Brief summary: stem cell types

ESC (embryonic stem cells): Pluripotent self-renewing stem cells derived from the inner cell mass of embryos
IPS (inducible pluripotent stem cells): Adult somatic stem cells derived from normal adult tissues modified through genetic engineering; They resemble pluripotent stem cells and have self-renewing potential
NSC (neural stem cells): Self-Renewing cells capable of differentiating into the most relevant brain cell types (neurons, astrocytes, oligodendrocytes)
BMSCs (bone marrow stem cells)
HSCs (hematopoietic stem cells, CD34+). Heterogeneous populations of multipotent cells capable of differentiating into all blood cell types (both myeloid and lymphoid)
EPCs (endothelial progenitor stem cells, CD34+). Circulating blood cells capable of differentiating into endothelial cells (angiogenesis)
MSCs (Bone Marrow Mesenchymal Stem Cells, CD34−). Multipotent stem cells from circulating blood with recently discovered reparative potential in damaged tissues.
MSC (mesenchymal stem cells)
ASC (adipose-derived MSCs). Mesenchymal stem cells highly concentrated in adipose tissues
pMSC (placental MSCs). Mesenchymal stem cells from the placenta
UCBs (umbilical cord blood MSCs). Mesenchymal stem cells in umbilical cord blood

### Experimental animal models

In experimental animal models, neural stem cell (NSC) administration has been shown to enhance axonal sprouting and transport, dendritic activity and the expression of neurogenesis, gliogenesis and neurotrophic support-associated genes [78–81]. While infarct size is not significantly reduced, levels of cell death and Bax-positive cells are decreased after 7 days of treatment in these experimental animals while Bcl-2 expression in the penumbra is augmented and neurological function is improved [82]. This is also observed when using other non-neural stem cell sources such as bone marrow (BMSC), umbilical cord blood cells (UCBC) or mesenchymal stem cells (MSC). Indeed, bone marrow mononuclear cell (BMMC) transplantation can promote proliferation of the endogenous NSCs and this is observed concomitantly with increased proliferation of endothelial cells (angiogenesis) following ischaemic stroke [83]. Endogenous NSC can be found around the peri-infarct area adjacent to endothelial cells, so it has been suggested that at least some NSCs are originated from microvascular pericytes. The mechanisms involved in the endogenous neurogenesis and vasculogenesis after BMC administration are still unclear and therefore under investigation.

Meanwhile, bone marrow-derived MSC [84] also hold great promise for cell therapy. The beneficial effects of MSC administration in experimental animal stroke models is well-described and there are a variety of studies with similar good results in structural/functional recovery [73, 85, 86]. A recent review summarizes the role of therapeutic mobilization of transplanted bone marrow stem cells and its importance for brain plasticity and remodelling in stroke [87]. Adipose tissue like bone marrow, is another source of MSC in which interest is growing because it provides an abundant, ethically unproblematic and accessible source of cells with similar potential to that of other adult stem cells [88, 89]. The same can be said of placenta cells, which also have low immunogenic properties and are easily obtained [90, 91]. *In vivo*, bioactive molecules secreted by MSCs provide a regenerative microenvironment that enhances a self-regulated regenerative response. This regenerative microenvironment (trophic activity) mediates tissue repair and regeneration under ischaemia conditions [92].

As was previously mentioned, endothelial cell regeneration and neovascularization after tissue ischaemia are subjects of interest nowadays in the context of brain repair and it has been reported that repair can be enhanced by the administration of endothelial progenitor cells (EPCs), the positive effects of which have been observed in long-term neurobehavioural tests [93, 94].

Lastly, another ethically unproblematic source of cells with great potential are inducible pluripotent stem cells (iPS). First described by the Yamanaka group [95], this kind of approach, combined with transplantation onto biodegradable matrices could provide an interesting framework for stem cell-based therapies [27]. In previous reports, iPS treatment has been shown to improve motor function, reduce infarct size, attenuate inflammatory cytokines and mediate protection [96]. However, as a therapeutic option, iPS cells require further evaluation in light of their high tumourigenic potential under certain conditions, a major concern for clinical use [67, 97].

Independently of the above-mentioned cell sources, an important practical issue is the type of transplant: autologous (same individual), allogenic (same species) or xenogenic (another species). To prevent rejection, autologous administration can be considered the best option. A limitation of this approach in a clinical situation is that it would only allow treatment several weeks after the stroke, as this is the time needed for the cultivation and expansion of cells from the donor [98]. However, as the most appropriate time for administering stem cells is not clear and pre-clinical data also indicate that acute allogenic administration is both safe and effective [99], it might be possible to consider the creation of biobanks of allogenic stem cells (donors) to treat cerebral infarct patients earlier, within the acute phase time period.

To emphasize results from a clinical perspective, the route of administration for stem cells is still a major concern. There are several possible options which have been tested in experimental animals including intrastriatal, intraventricular, intravenous [100], intracarotid [101] or intranasal routes [102]. Some of them have an apparently similar effectiveness, but intravenous administration would be the least invasive delivery mode for use in future clinical applications [99]. However, as stroke is a localized CNS disease, new options and ideas in CNS-directed delivery are desirable. Also, new implantation sites such as the epi-cortical implant, a new minimally invasive method [103] or also the plexus-CSF route [104], would minimize or eliminate the distribution of graft cells to peripheral organs and obviate the need for a surgical (cell) implantation that is required by the intracarotid route. Either way, studies in experimental animals should focus on imaging and cell tracking of the transplanted cells.

Taking into consideration that in patients acute stroke is usually considered a ‘time is life’ condition, the time of transplant is critical, and, for now, there is no clear concordance between animal models and humans. It has been described that the blood-brain barrier is open continuously for several weeks after ischaemia [105], indicating that the injured tissue may permit the entrance of exogenous cells during a long post-ischaemic window and this possibility has resulted in most studies having been focused on post-acute MSC administration [73]. However, with these long experimental conditions it is not possible to evaluate the protective effects, if any, of these cells and whether early administration might interact with reparative modulation in the brain. Furthermore, gliogenesis could also have a detrimental role as glial scarring in the late state of cerebral infarct may impede or compromise the delivery of new cells to the peri-infarct areas where they could exert their positive function.

### Clinical studies

Globally and in contrast to results from experimental animal models (Table 3), clinical trials with stem cells have reported safety but mixed results in terms of efficacy [106]. As an example, cultured human (h) NSCs stereotactically implanted in patients with motor deficits, did not produce evidence of a significant benefit in terms of motor function although safety and feasibility was confirmed [107].

**Table 3:** Main results of stem cells in animal models and cerebral infarct clinical trials

	Animal models	Clinical trials
Stem cells		
Neural stem cells (NSCs)/neuronal cells	Promotes behavioural recovery and endogenous neurogenesis [79], reduces infarct volume [80] enhances axonal sprouting and the expression of genes involved in neurogenesis, gliogenesis, and neurotrophic support; modulates microglial response [118]. Anti-apoptotic activity [82]	Phase II (18 patients): No evidence of a significant benefit in motor function but safety and feasibility demonstrated in [107] Safety of a manufactured neural stem cell line (CTX0E03) is being tested (PISCES study, Phase I)
Mesenchymal stem cells (MSCs)	Enhances structural/functional recovery [85], reduces lesion volume, decreases inflammatory cell proliferation [86, 88]	Stereotactic implantation: Safety: Open study (5 patients): with excellent tolerance [108] Intravenous administration: Safety: Open label (12 patients): no safety concerns [109] Safety and efficacy: Phase I/II (30 patients) no adverse events and better outcomes in MSC-treated patients [98] Open label long-term follow-up (52 patients): safe and clinical improvement [110]
Bone marrow stem cells (BMSCs)	CD34: enhanced neovascularisation, neurogenesis, functional recovery [119] EPCs: protected the brain against ischaemic injury, promoted neurovascular repair and improved long-term neurobehavioural outcomes [93]	Safety: Ongoing Phase I and Phase II trials. CD34: autologous CD34+ subset BMSC infusion and intercerebral implantation

Main results of stem cell therapy in animal models and clinical trials of cerebral ischaemia. The reference number for each study is shown in brackets. Information from ongoing clinical trials can be seen in the PubMed (http://www.ncbi.nlm.nih.gov/pubmed/) and Clinical trials (http://clinicaltrials.gov/) databases.

With regards to MSCs, some studies have reported that they can be safely transplanted into the brain of patients with excellent tolerance and without complications [108]. Also systemically, in patients with severe cerebral infarcts, intravenous infusion of autologous MSCs appears to be a feasible and safe therapy [109] that may improve functional recovery [98]. A long-term follow-up study has shown the safety of the treatment after 5 years [110], this kind of positive result is interesting and the study should be replicated with larger cohorts of patients.

Currently, there are open clinical trials using MSCs in ischaemic stroke. Phase I and phase II studies exist for BMMCs and results will be obtained soon. Other studies are evaluating the feasibility and tolerance of the intravenous injection of autologous MSCs in phase II and another 2 clinical trials in phase I/II are evaluating the intravenous injection of allogenic MSCs 60. There are considerable difficulties in designing future efficacy trials, some of which are inherent to the field of regenerative treatment in stroke, and others specific to stem cells or their mode of delivery [111].

As has been discussed above, multiple subtypes of stem cell therapies have been developed in recent years for the treatment of cerebral ischaemia. However, large and well-designed trials are needed to identify the best options for their transfer to the clinical setting [112]. In 2007, the Stem Cell Therapy as an Emerging Paradigm for Stroke (STEPS) meeting was organized to accelerate the field of cell therapy for stroke and to address outstanding questions [113]. In 2010, a second meeting, STEPS2, was held. Participants identified critical gaps in knowledge and research areas that require further studies, updated existing guidelines and drafted new recommendations to create a framework to guide future investigations into cell-based therapies for stroke [114]. In summary, larger trials with stringent and well-delimited inclusion criteria are necessary as the results from pre-clinical studies still support significant beneficial effects from cell therapy. Furthermore, a better understanding of cell fate following infusion in patients is desirable.

In our opinion, as suggested in a recent review by our group [115], and based on the lack of expression of MHC class II antigens, the use of allogenic mesenchymal stem cells [99] may broaden therapeutic interest in their use. This type of cell has been shown to be a good alternative for treating patients with cerebral infarction in the acute phase. Also the IV administration route is the least invasive and may offer the most suitable strategy for its clinical translation. Their administration during the acute phase could help to inhibit the first steps of the ischaemic cascade after stroke and enhance endogenous mechanisms of brain repair. Therefore, for the authors, the IV administration of mesenchymal stem cells in the acute phase amplifies the resources for good functional recovery and may be an effective therapy in the future.

## Last comments

In cerebral ischaemia, protection and brain repair mechanisms are activated and orchestrated as a continuum once the disease process is initiated. Both trophic factors and stem cell therapy have been shown to modulate genetic and molecular programs underlying neuronal cell survival and axonal connectivity, angiogenesis, oligodendrogenesis and modulation of inflammation. Both therapeutic approaches have consistently shown exciting results in experimental animal models but their efficacy in patients has not yet been confirmed. Although not entirely understood, recent data have demonstrated paracrine interactions between stem cells and trophic factors, and this could suggest a multi-modal strategy for brain repair. Experimental research in the coming years will be focused on combining both approaches. At the same time, protocolizing the clinical context, dose, times and routes of administration will help design more effective clinical trials.
